# Genetic Forms of Calciopenic Rickets

**DOI:** 10.5152/eurasianjmed.2022.22322

**Published:** 2022-12-01

**Authors:** Ayse Sena Donmez, Ayberk Turkyilmaz, Atilla Cayir

**Affiliations:** 1 Department of Pediatrics, Regional Training and Research Hospital, Erzurum, Turkey; 2 Department of Medical Genetics, Karadeniz Technical University Faculty of Medicine, Trabzon, Turkey; 3 Department of Pediatric Endocrinology, Regional Training and Research Hospital, Erzurum, Turkey

**Keywords:** Vitamin D deficiency/diagnosis, mutation, rickets/genetics, *VDR*, *CYP27B1*

## Abstract

Rickets is a disease involving calcium and phosphate balance disturbances in the pediatric population. A series of hereditary disorders known as vitamin D-dependent rickets are defined as early-onset rickets resulting from either an insufficient response to active vitamin D or an inability to maintain adequate levels of the active forms of vitamin D. According to the age at onset and the pathophysiology of the disease, various clinical signs including growth failure, limb bowing, and joint enlargement may be present. Vitamin D-dependent rickets type 1A, type 1B, type 2A, type 2B, and type 3 are classified as genetic forms. Further studies are crucial for the development of targeted therapies and future mutation-specific therapies.

**Main Points**

- Rickets is a significant childhood illness that persists despite vitamin D supplementation programs.
- If there are signs and symptoms of rickets despite vitamin D supplementation, clinicians should consider the possibility of vitamin D-dependent rickets.
- The discovery of new mutations that cause hereditary types of rickets is leading to a better understanding of the underlying mechanisms and new therapeutic opportunities.

## Introduction

As a fat-soluble vitamin, vitamin D is crucial for the metabolism of calcium and phosphate.^1-3^ The body converts cholecalciferol, which is produced in the skin upon exposure to the sun or consumed via food but is not biologically active, into active vitamin D through 2 hydroxylation reactions.^4,5^ The liver is the site of the initial hydroxylation process. The enzyme 25-hydroxylase in the liver converts it to 25-OH vitamin D [25(OH)D or calcidiol]. The 1-hydroxylase enzyme produced by the kidneys causes 25(OH)D to go through a second hydroxylation. This transforms it into calcitriol [1,25(OH)_2_D], an active metabolite of vitamin D.^6,7^ By attaching to the vitamin D receptor (VDR) and vitamin D binding protein, 1,25(OH)_2_D becomes functional in target cells.^8-12^ It is stored in adipose tissue, muscles, and the liver.^13^

Rickets, previously known as rachitis, is a disease characterized by inadequate mineralization of growth plate cartilage and the bone matrix in bone tissue during the period when the growth plates are open.^14,15^ Rickets, which is more common in developing countries, may be secondary to different etiologies with nutritional, genetic, and drug-based factors or prematurity.^16-18^ It is an important cause of morbidity and may lead to delayed growth and motor development, short stature, skeletal deformities, tetany, and seizures.^19^

Rickets can be classified into 3 main categories. Calciopenic rickets occurs as a result of vitamin D metabolism disturbance or inadequate dietary calcium intake; phosphopenic rickets is a result of dietary phosphate deficiency, impaired intestinal phosphate absorption, or disorders of phosphate metabolism that cause excessive loss of phosphate from the kidneys; rickets with mineralization inhibition is caused by hereditary hypophosphatasia, fluoride/aluminum toxicity, and the use of first-generation bisphosphonates.^20-22^

Roughly 13% of all cases of rickets are due to hereditary factors. There are 2 main categories of hereditary rickets, and vitamin D-dependent rickets (VDDR) occurs in the gene encoding the vitamin D receptor or as a result of gene abnormalities that affect the encoding of enzymes involved in vitamin D production. More VDDR cases occur in underdeveloped countries.^23^ On the other hand, due to genetic damage, hypophosphatemic rickets is linked to abnormalities in renal phosphate transport or reabsorption.^24,25^

**Figure 1.**
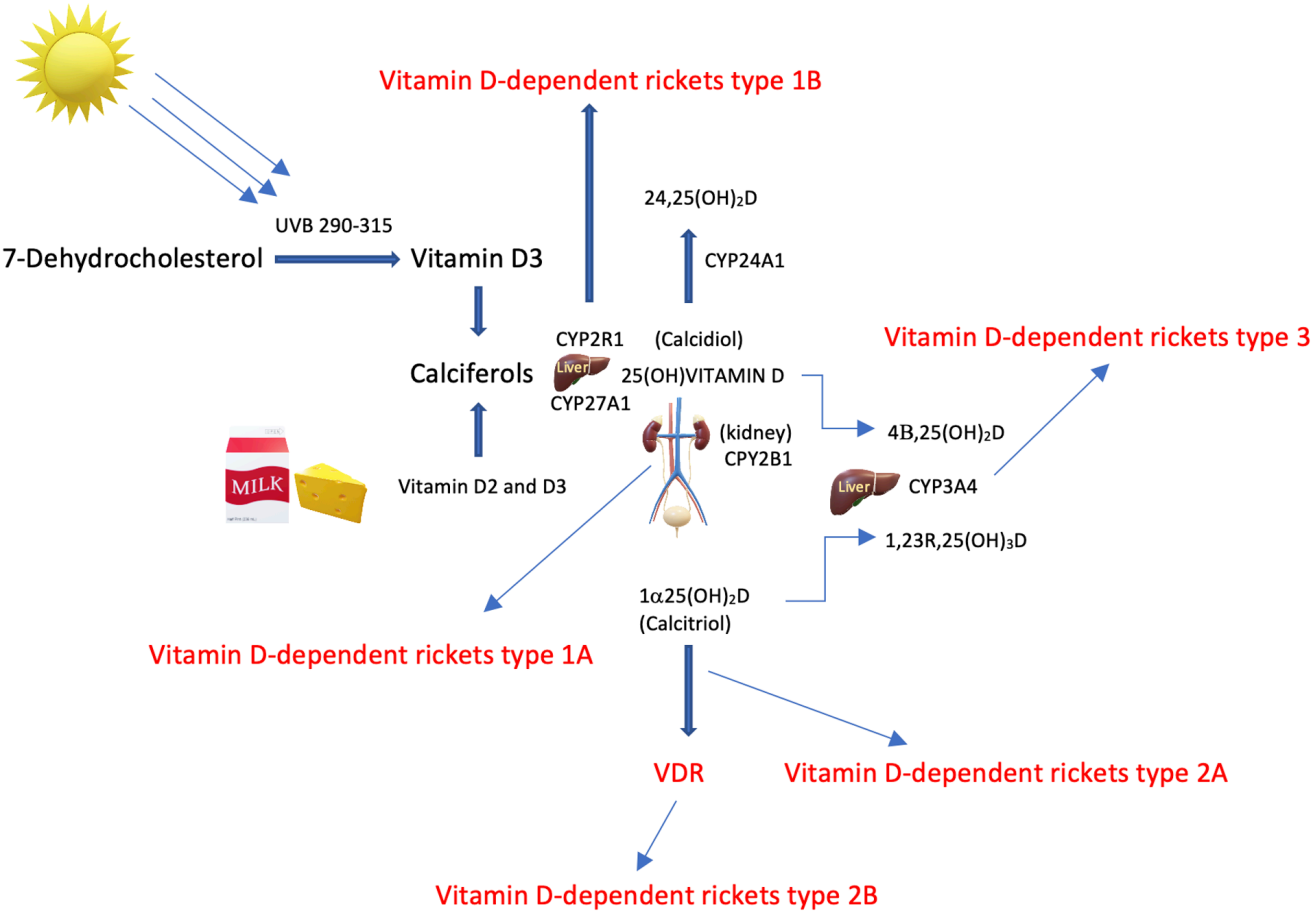
Hereditary forms of calciopenic rickets and vitamin D homeostasis.

Vitamin D-dependent rickets types 1A and 1B, which are defined by vitamin D insufficiency, and types 2A and 2B, which are characterized by vitamin D resistance, constitute 2 main subgroups of hereditary calciopenic rickets (Figure 1). The clinical and laboratory results reported in cases of hereditary calciopenic rickets are summarized in Table 1.^26,27^

## Vitamin D-Dependent Rickets Type 1A (MIM 264700)

The production of vitamin D and its conversion to an active state are both primarily carried out by the enzyme 1-hydroxylase. An inactivating mutation in *CYP27B1*, which encodes 1-hydroxylase, causes VDDR1A.^28^ This mutation prevents 25(OH)D from converting into 1,25(OH)_2_D, which results in rickets.^29^

### Clinical Findings

Clinical findings are similar to those of nutritional rickets. Hypocalcemic seizures, hypotonia, growth retardation, and muscle weakness may be observed in early infancy.^30,31^ 25(OH)D deficiency has been reported to affect the muscles of the lower extremities, which are especially crucial for walking and postural balance.^32^ Patients are usually clinically normal at birth, but severe signs of rickets may appear in the first 2 years of life.

### Laboratory Findings

Laboratory findings seen in VDDR1A are as follows^25,26,33^:

- Low serum calcium
- Low/normal serum phosphorus
- High parathyroid hormone (PTH)
- Elevated alkaline phosphatase
- Normal serum 25(OH)D
- Low serum 1,25(OH)_2_D: The 1,25(OH)_2_D levels in VDDR1A are typically low from birth and do not rise with vitamin D therapy.^34,35^ Parathyroid hormone secretion and elevated PTH levels are brought on by deficiencies of vitamin D, calcium, and ionized calcium.^36^ In some circumstances, otherwise normal 1,25(OH)_2_D levels with hypophosphatemia, hypocalcemia, and elevated PTH levels should be regarded as low.^37^
- Normal or elevated cyclic adenosine monophosphate (cAMP) in urine
- Increased intestinal calcium excretion
- Normal 24,25(OH)_2_D level, that is, normal 24-hydroxylase enzyme activity.

### Treatment

Calcitriol, the first-choice treatment, rapidly corrects the clinical, biochemical, and radiological findings of VDDR1A at physiologic doses. The goal of treatment is to increase calcium levels and other biochemical parameters to normal ranges without hypercalciuria, improve radiological findings, increase muscle strength, and ensure normal growth and development.

Calcitriol is started at 10-20 ng/kg/day in 2 doses or 1-2 µg/day, and treatment can be continued at a dose of 0.5-1 µg/day in follow-up. Alfacalcidol can also be used at a dose of 1-3 µg/day. Adding 30-75 mg/kg/day of elemental calcium to calcitriol or alfacalcidol should be recommended at the beginning of treatment. Lifelong treatment is required for patients with VDDR1A. With adherence to treatment, biochemical parameters normalize and adult height with normal linear growth and normal bone mineralization can be achieved.^25,26,38^

## Vitamin D-Dependent Rickets Type 1B (MIM 600081)

A rare autosomal recessive condition, VDDR1B is brought on by homozygous or compound heterozygous mutations in the *CYP2R1* gene (cytochrome P450, family 2, subfamily IIR, polypeptide 1; MIM 608713).^39,40^ In patients presenting with signs of rickets, low 25(OH)D levels as also seen in cases of nutritional rickets may cause difficulty in diagnosing VDDR1B. This type of rickets should be considered for patients who have no signs of malabsorption and for whom standard vitamin D doses do not result in an adequate increase in 25(OH)D levels. These patients generally have a milder phenotype compared to those with VDDR1A.^26,41^

### Clinical Findings

Clinical findings are as in VDDR1A (Table 2).^42^

### Laboratory Findings

Laboratory findings of VDDR1B are as follows^26,43^:

- Low/normal calcium
- Low/normal phosphorus
- High alkaline phosphatase
- High PTH
- Normal serum 1,25(OH)_2_D
- Low serum 25(OH)D with insufficient elevation despite standard-dose vitamin D therapy.

### Treatment

Treatment may include high doses of vitamin D.^44^ There is no standard regarding the dose of vitamin D to be given. In the literature, oral administration of 4000-10 000 U/day or 50 000 U/month intramuscularly for 6 months has been reported. Calcitriol can be used at 10-20 ng/kg/day with 2 oral doses. Patients receiving calcitriol treatment should be followed for the risk of hypercalciuria or nephrocalcinosis. There is insufficient information in the literature regarding long-term treatment results.^26,43,45^

### Vitamin D-Dependent Rickets Type 2A (MIM 277440)

Vitamin D-dependent rickets type 2A is an inherited autosomal recessive type of rickets characterized by resistance to 1,25(OH)_2_D.^46^ It is also called vitamin D-resistant rickets, hereditary 1,25(OH)_2_D-resistant rickets, rickets-alopecia syndrome, hereditary hypocalcemic vitamin D-resistant rickets, and pseudovitamin D-deficiency type 2A.^7,26^

### Clinical Findings

Clinical findings are the same as those seen with VDDR1A in addition to total or partial alopecia seen in the majority of cases (Table 2).^42^ The clinical findings are heterogeneous. Disease manifestations usually occur in infancy or early childhood, but sporadic late-onset cases with milder clinical manifestations may also be seen. Severe signs of rickets such as convulsions, hypotonicity, and growth disorders are often seen in the first years of life.^7,47-48^

In contrast to other forms of rickets, more than 50% of patients with VDDR2A have sparse body hair and alopecia from birth or the first years of life.^49^ In patients with alopecia, signs of rickets appear earlier and the clinical course is more severe. The extent of alopecia ranges from hair sparseness to the absence of eyelashes and eyebrows or total alopecia.^49^ The absence of alopecia in VDDR1A and VDDR1B indicates that calcitriol receptor function rather than vitamin D plays a role in hair follicle development. In some severe cases, patients may die in the first year of life with hypocalcemic convulsions and pulmonary complications.^50,51^

### Laboratory Findings

Laboratory findings in cases of VDDR2A are as follows^51-53^:

- Low calcium
- Low phosphorus
- High alkaline phosphatase
- High PTH
- Normal 25(OH)D
- Serum 1,25(OH)_2_D level 3-30 times higher than normal (300-1000 pg/mL)
- Low 24,25(OH)_2_D levels are caused by hypocalcemia, hypophosphatemia, and elevated PTH, respectively, activating 1-hydroxylase and blocking 24-hydroxylase.

### Treatment

There is no standard treatment protocol for patients with VDDR2A. It is advised to administer elemental calcium at a dose of 1-3 g/day and calcitriol orally at 1-6 µg/kg/day.^8,20^ Recommended daily doses are 5000-40 000 IU for vitamin D, 20-200 g for 25(OH)D3, and 17-20 g for 1,25(OH)_2_D3.^27^ Depending on the specific *VDR* mutation, responses to medication may change. High doses of elemental intravenous calcium are needed for resistant patients who do not react to vitamin D therapy, such as 400 to 1400 mg/m^2^/day.^54^ For the majority of patients with VDDR2A, extended hospitalization with intravenous calcium treatment may be required to achieve normocalcemia.

## Vitamin D-Dependent Rickets Type 2B (MIM 600785)

Vitamin D-dependent rickets type 2B is an uncommon condition. The clinical and laboratory findings are comparable to those of VDDR2A, but the *VDR* gene is unaltered. The molecular etiology is still not completely understood. Vitamin D receptor functions and VDR-RXR heterodimer formation were shown to be normal in contrast to VDDR2A patients.^55^

Alopecia may accompany clinical and laboratory findings in VDDR2B that are comparable to those of VDDR2A.^56^ It is possible to distinguish between VDDR2A and VDDR2B by genetic testing. These patients are treated similarly to VDDR2A patients.^25,26^

## Vitamin D-Dependent Rickets Type 3

Increased inactivation of vitamin D metabolites results in VDDR3. This is induced by a p.I301T mutation in the *CYP3A4* gene.^57^ Clinically and experimentally, it is comparable to VDDR1.^58^ It has been indicated that high-dose vitamin D therapy is beneficial.^54^ However, there is only one clinical trial in the literature for VDDR3.^57^ Further studies are crucial for the future development of targeted therapies and mutation-specific therapies.

## Figures and Tables

**Table 1.** Clinical and Laboratory Findings in Vitamin D-Dependent Rickets (VDDR)

	VDDR1A	VDDR1B	VDDR2A	VDDR2B	VDDR3
Age at onset of symptoms	Infant/child	Infant/child/puberty	Infant/child	Infant/child	Infant/child
Inheritance	Autosomal recessive	Autosomal recessive	Autosomal recessive	Unknown	Unknown
Genetic defect	CYP27B1 mutation	CYP2R1 mutation	VDR mutation	HNRNPC Overexpression	CYP3A4 mutation
Pathophysiology	1-alpha hydroxylase	25-hydroxylase	Vitamin D receptor	Heterogeneous nuclear ribonucleoprotein C1, C2 protein	CYP3A4 gain-of-function mutation
Original finding	-	-	Alopecia	Alopecia	-
Serum calcium	↓/N	↓/N	↓/N	↓/N	↓/N
Serum phosphorus	N/↓	N/↓	N/↓	N/↓	N/↓
Alkaline phosphatase (ALP)	↑	↑	↑	↑	↑
Parathyroid hormone	↑	↑	↑	↑	↑
25(OH)D	N/↑	↓	N	N	↓
1,25(OH)_2_D	↓/N	N/↓	↑	↑	↓
Urine calcium	↓	↓	↓	↓	↓

**Table 2.** Clinical Features of Vitamin D-Dependent Rickets Type 1A

**Findings related to the skeletal system** - Expansion of the fontanelle - Delayed fontanelle closure (normally by 2 years of age) - Craniotabes* - Caput quadratum - Frontal bossing - Tooth eruption is delayed (incisors have not erupted by 10 months of age and molars by 18 months of age) - Early tooth decay due to gingival hypoplasia - Swelling of the wrists and ankles due to metaphyseal enlargement of the bones* - Rachitic rosary, prominent lateral to the breast line as a result of enlargement of the costochondral junction* - Harrison sulcus - Chest wall deformities such as pectus carinatum, pectus excavatum, or narrow rib cage - Leg deformities such as genu varum, genu valgum, or windswept deformities in the tibia or femur - Bone pain	**Nonskeletal findings** - Widespread and severe muscle weakness, especially prominent in proximal and thoracic muscles - Delays in motor development and motor functions such as head control, sitting without support, standing, and walking - Restlessness, irritability - Tetany, convulsions, and laryngospasm due to hypocalcemia - Hypocalcemic dilated cardiomyopathy including heart failure, arrhythmia, sudden cardiac arrest, and death - Lung infections and atelectasis due to thoracic deformities and muscle weakness - Delayed growth and development - Increased intracranial pressure	**Radiological findings** - Enlargement in the area between the metaphysis and epiphysis (the earliest findings in infants are in the distal ulnar region) - Irregularity of the metaphyseal border, brushy-edged appearance of the metaphysis, cupping, diffuse loss of mineralization, and osteopenia - Increased trabeculation - Cortical thinning - Bending of bones - Subperiosteal erosion - Pelvic deformities such as narrowing of the pelvic outlet - Spinal cord deformities such as scoliosis - Pathological fractures

*Most sensitive physical examination findings.
